# Oxidative stability of sunflower and soybean oils enriched with black plum peel extract in comparison with synthetic antioxidants

**DOI:** 10.1371/journal.pone.0279735

**Published:** 2023-01-20

**Authors:** Mohaddeseh Kariminejad, Abolfazl Naimabadi, Afsaneh Morshedi, Toktam Mohammadi-Moghaddam, Abolfazl Shokuhi, Mahsa Bordbar

**Affiliations:** 1 Department of Food Science and Technology, Neyshabur University of Medical Sciences, Neyshabur, Iran; 2 Department of Environmental Health Engineering, School of Public Health, Neyshabur University of Medical Sciences, Neyshabur, Iran; 3 Department of Food Science and Technology, Ferdowsi University of Mashhad, Mashhad, Iran; 4 Student Research Committee, Neyshabur University of Medical Sciences, Neyshabur, Iran; Universiti Teknologi Malaysia, MALAYSIA

## Abstract

Black plum peel is the by-product of plum processing and is a valuable source of antioxidants and phenolic compounds. In this research, total phenolic compounds, total flavonoid content and antioxidant activity of black plum peel were measured. After that, black plum peel extract (in concentrations 0, 400, 800, 1200 and 2000 ppm) as a natural antioxidant for improving the stability of soybean and sunflower oil was used. The oxidative stability parameters of oils (peroxide value, free fatty acids, thiobarbituric acid, conjugated dienes, and carbonyl value) were measured at 60 °C for 4–16 days. Antioxidant activity, total phenolic compounds and total flavonoid content of black plum peel were 86.87% and 100.53 mg GA /g and 871.062 mg Quercetin/g respectively. Black plum peel extract could have a significant positive effect (P<0.05) on improvement of the quality and stability parameters of soybean oil and sunflower oil. The oxidative stability parameters for commercial oils and samples containing black plum peel extract were near each other and in an acceptable range. So, black plum peel is recommended as an oxidative stabilizer of oils and alternative synthetic antioxidants.

## Introduction

One of the series reactions that could happen during processing, storage and final preparation in oil and some foods which contain lipids, is an oxidative reaction [1–3]. This reaction regularly starts immediately after oil extraction which not only could affect unpleasant flavor, rancid odor and discoloration of oil or food but could also alter the quality and shelf life of food [1, 2, 4, 5]. Auto-oxidation, photo-oxidation, ketonic and enzymatic oxidation are kinds of these deteriorative reactions which auto-oxidation being the most common [6]. Auto-oxidation is a reaction that takes place between un-saturated fatty acid and oxygen via an autocatalytic process which includes free radicals mechanism, peroxide-free radicals and hydro-peroxides [6, 7]. Some factors that accelerate auto-oxidation are the presence of oxygen, unsaturation, moisture, temperature, light and heavy metal, that the most important factor is the unsaturation of oil [1, 6]. Peroxide is the primary product of oil oxidation processes and a higher value than 9 meqO2/kg indicates oil oxidation [8]. In general, the lower the peroxide value, the better the quality of the oil [9]. The presence of peroxide in the oil as a catalyst accelerates oxidation, so measuring the peroxide index is one of the indicator tests used to determine the degree of oil spoilage [10]. Among edible oils, Soybean oil (SBO) and Sunflower seed oil (SFO) are two of the popular vegetable oils which are mainly used in the pharmaceutical, cosmetic and food industries. Both of them are required and necessary in the human body due to the rich sources of essential fatty acids and liposoluble vitamins [11]. SFO as one of the important edible and unsaturated oils includes a high amount of linoleic acid (18:2 ω-6) that is polyunsaturated and an essential fatty acid [4, 12]. SBO is contained a high level of α-linolenic acid (18:3 ω-3) [13, 14]. Both SBO and SFO are very sensitive to oxidation, because of the unsaturated fatty acids that they have in their compositions [11].

In order to prevent oxidation reaction mechanism in edible frying oils, synthetic antioxidants, t- butyl hydroquinone (TBHQ), butylated hydroxyanisole (BHA) and butylated hydroxytoluene (BHT), are widely used in the food industry [3, 4, 15]. In spite of the high efficiency, high stability and low cost of synthetic antioxidants in food products, recently, awareness about the toxic and carcinogenic effect of synthetic antioxidants on the human body is increased [16–19]. Thus, there is a tendency to use natural antioxidants as many researchers have studied plant sources because of their much containing of antioxidant activity and phenolic compounds that could be extracted [1, 3–6, 9, 10, 12, 15, 20–23]. Antioxidant activity and phenolic compounds could be really effective to prevent oxidation reactions as natural antioxidants [24].

Plum (*Prunus subg*. *Prunus*) is one of the important fruits in Iran and it’s a rich source of carbohydrates, amino acids, vitamins, minerals, dietary fibers and phenolic compounds. During the processing of plum fruits, especially drying, plum peel is remained as a by-product. The moisture content of this material is high and it is susceptible to microbial spoilage. Most of the time, it is left in the environment and causes environmental pollution. According to the FAO (2020) the production of plum in Iran in 2018 was 313,103 tons that 20% of it is consumed fresh and 80% is processed [25, 26]. In general, one-fifth of the weight of a plum is peel. So, more than 50,000 tons of plum peel was produced in Iran [26]. So far, no application has been made for this material, while, it has all the functional properties of the plum fruit and could be used in the food industry. The amount of antioxidants and total phenolic compounds in black plum peel puree has been reported at 88.59% and 105.91 mg/g GA, respectively [27]. It seems that black plum peel can use as a natural antioxidant to inhibit oxidation and eliminate free radicals. Literature review showed there are different researches on the usage of fruits and vegetables extract as natural antioxidants for the stability of edible oils [28–33]. There is no research available concerning the usage of black plum peel extract as an antioxidant in edible oils. So, the aim of this study was to measure total phenolic compounds, total flavonoid content and antioxidant activity of black plum peel and to investigate the effect of black plum peel extract (in concentrations 0, 400, 800, 1200 and 2000 ppm) as a natural antioxidant on oxidative stability parameters (peroxide value, free fatty acids, thiobarbituric acid, conjugated dienes and carbonyl value) during the storage time (60 °C for 4–16 days) of SBO and SFO in comparison with oils that contain industrial antioxidants.

## Materials and methods

### Preparation of black plum peel extract (BPPE)

After washing and removing impurities, the plum peel was crushed in an industrial crusher and was kept in the freezer until experiments. For the preparation of BPPE, about 10 g of BPP was dissolved in 100 ml of 80% ethanol and placed at 50° C for 18 to 24 hours. After that, it was filtered with Whatman filter paper 1 and Buchner funnel. The solvent was evaporated and black plum peel extract (BPPE) was kept at -18° C until the experiments.

### Determination of total phenolic compounds of BPPE

In order to determine the total phenolic compounds, Folin–Ciocalteu reagent was used [1]. A total of 3 ml distilled water was added to 50 μl of BPPE. Then 250 ml of Folin–Ciocalteu reagent was added to the solution. After mixing 750 μl of carbonate sodium and 950 μl of distilled water, the mixture was shaken and allowed to be put in a dark room at room temperature for 15 minutes. The solution absorbance was measured at 760 nm. This test was repeated three times and the results were calculated by a calibration curve plotted with gallic acid as μg gallic acid per gram dry sample.

### Determination of total flavonoid content of BPPE

The total flavonoid content of BPPE was evaluated according to Moo-Huchin et al. 2015 [34]. A total of 1 ml BPPE sample was mixed with 300 μl of NaNO_2_ 5% and 4 ml of distilled water. After 5 minutes, 300 μl of ALCl_3_ was added to the mixture. Then, NaOH 1M was added to the mixture and the absorbance was measured at 415 nm. The concentration of total flavonoids of BPPE was calculated by the standard curve of quercetin.

### Determination of antioxidant activity of BPPE

The antioxidant activity of the sample was evaluated according to the DPPH method reported by Mezza et al. 2018 [12]. A total of 3 ml ethanolic solution of DPPH reagent was added to the sample and allowed to place a darkroom and room temperature for 1 hour. Then, the UV absorbance was determined at 517 nm. The results were calculated using Eq 1:

$$DPPH\;scavenging\;activity(\%)=\frac{Abs\left(DPPH\right)-Abs(extract)}{Abs\left(DPPH\right)}\times 100$$ (1)

### Preparation of oil samples

SBO and SFO were obtained from the Shadgol industry, Neyshabur, Iran. BPPE was added to SBO and SFO in four different concentrations (400, 800, 1200 and 2000 ppm) as antioxidants. Samples were stored at 60°C for 16 days and their quality characteristics contain peroxide value (PV), free fatty acids (FFA), thiobarbituric acid (TBA), conjugated dienes (CD) and carbonyl value (CV) were measured at 4, 8, 12 and 16 days. In the end, the quality parameters of SBO and SFO were compared with the same brand of oils that contain commercial antioxidants.

### Peroxide value (PV) determination

PV of oil samples was determined according to Delfanian et al. 2016 [1]. At first, 0.2 mg of each oil was mixed with Chloroform methanol. Then 50 μl of Iron (II) chloride solution and 50 μl of Ammonium thiocyanate solution (30% w/v) were added to the mixture and were shaken for 2–4 seconds. After incubating the samples at room temperature, the absorbance was read at 500 nm. Results were expressed in Milli-equivalents of oxygen per kilogram of each sample.

### Free fatty acids (FFA) determination

The analysis of FFA was performed using a 2 mg neutralized sample that was neutralized with 50 ml of Ether ethanol (2:1, v/v) [4]. The mixture was shaken by hand and cooled to room temperature. The solution was titrated against Potassium hydroxide (KOH) and as an indicator, Phenolphthalein solution regent was used. To expose the FFA values, results were calculated according to Eq 2:

$$FFA\;(\frac{mg}{gr})=(V\times C\times 56.11)/m$$ (2)

where V is the value of KOH, C is the concentration of KOH, and m is the weight of the oil sample.

### ThiobarBituric Acid (TBA) value determination

TBA analysis was conducted using Afshari et al. 2017 [24]. 200 mg of each sample was dissolved in 25 ml of 1Butanol. After mixing, 10 ml of TBA (2%) was added to 5 ml of this solution and incubated for 2 h at 95°C. Then the mixture was placed in a water bath until 25°C. The absorbance was read at 532 nm. TBA was calculated using Eq 3:

TBA=5(A-B)/m (3)

where A is absorbance, B is the absorbance of the control sample and m is mg of oil sample.

### Conjugated Diene (CD) determination

The analysis of CD was performed using Delfanian et al. 2016 [1]. Approximately 5mg of oil samples were dissolved in 10 ml of Cyclohexane. The absorbance was measured at 233 nm.

### Carbonyl Value (CV) determination

CV was determined according to Delfanian et al. 2016 [1]. The calibration curve of standard aldehyde (2, 4-Decadienal) was drawn in a concentration range of 50–500 μM. A total of 50 mg of 2,4 D-nitrophenyl hydrazine (DNH) and 100 ml 2-Propanol were mixed together. Approximately 0.15 g of oil sample in a volumetric flask and filled with solvent contained 0.4 mg/ml of Triphenylphosphine (TPP). A total of 1 ml DNH solution was added to 1 ml of the prepared solution and kept at 40°C for 20 minutes. After that, the solution was cooled. In the next step, 8 ml of KOH (2%) was added and it was centrifuged for 5 minutes at 2000 rpm. The absorbance of the supernatant was numerated at 420 nm and the obtained results were reported in μmole of 2, 4-Decadienal per grams of each sample.

### Statistical analysis

The statistical analyses (ANOVA) were carried out by Minitab 16 (Minitab, Inc., State College, PA, USA) using a full factorial design. All experiments were performed in triplicate. Means were separated by Tukey analysis with at least a significant difference of *P* ≤ 0.05 value. GraphPad Prism (Version 8.0.1, USA) was also used to plot the curves.

## Results and discussions

### Comparison of SBO and SFO in terms of oxidative stability

Table 1 presents the experimental data of oxidative stability for raw and commercial SBO and SFO. From the data, for raw SBO, the amount of PV, FFA, CV and CD is significantly higher than commercial. No significant differences were found for TBA of raw and commercial SBO. As shown in Table 1, the amount of FFA, TBA and CD for raw SFO is significantly higher than commercial SFO. It was also observed that PV, CV and CD in SBO is more than SFO and FFA is less, which can be due to the different composition and amount of their fatty acids and the presence of linolenic acid in the composition of SBO. This finding is in agreement with [11] finding which showed the acid value and peroxide value for raw SBO were more than for raw SFO. Furthermore, they reported that SBO had more stability than SFO and the reason was the higher unsaturated fatty acids in SFO. According to the results of that research, Linolenic acid for SBO and SFO was 5% and 0.5% and saturated fatty acids were 15.5% and 10.7% for SBO and SFO, respectively.

**Table 1:** Oxidative stability parameters of raw and commercial soybean and sunflower oils.

Oil		PV	FFA	TBA	CV	CD
**Soybean**	**Raw**	*1.26±0.02^a^**	0.40±0.02^b^	0.25±0.01^a^	2.00±0.11^a^	1.65±0.11^a^
	**Commercial**	0.74±0.06^b^	0.22±0.01^c^	0.22±0.01^ab^	1.71±0.15b	1.25±0.03^d^
**Sunflower seed**	**Raw**	0.43±0.02^c^	0.81±0.06^a^	0.25±0.03^a^	1.52±0.20^c^	1.56±0.04^b^
	**Commercial**	0.39±0.01^c^	0.41±0.03^b^	0.21±0.02^b^	1.51±0.08^c^	1.35±0.01c

*Mean±SE.

**Letters show significance at 0.05.

### Total phenolic compounds, total flavonoid content and antioxidant activity of black plum peel (BPP)

**Fig 1:**
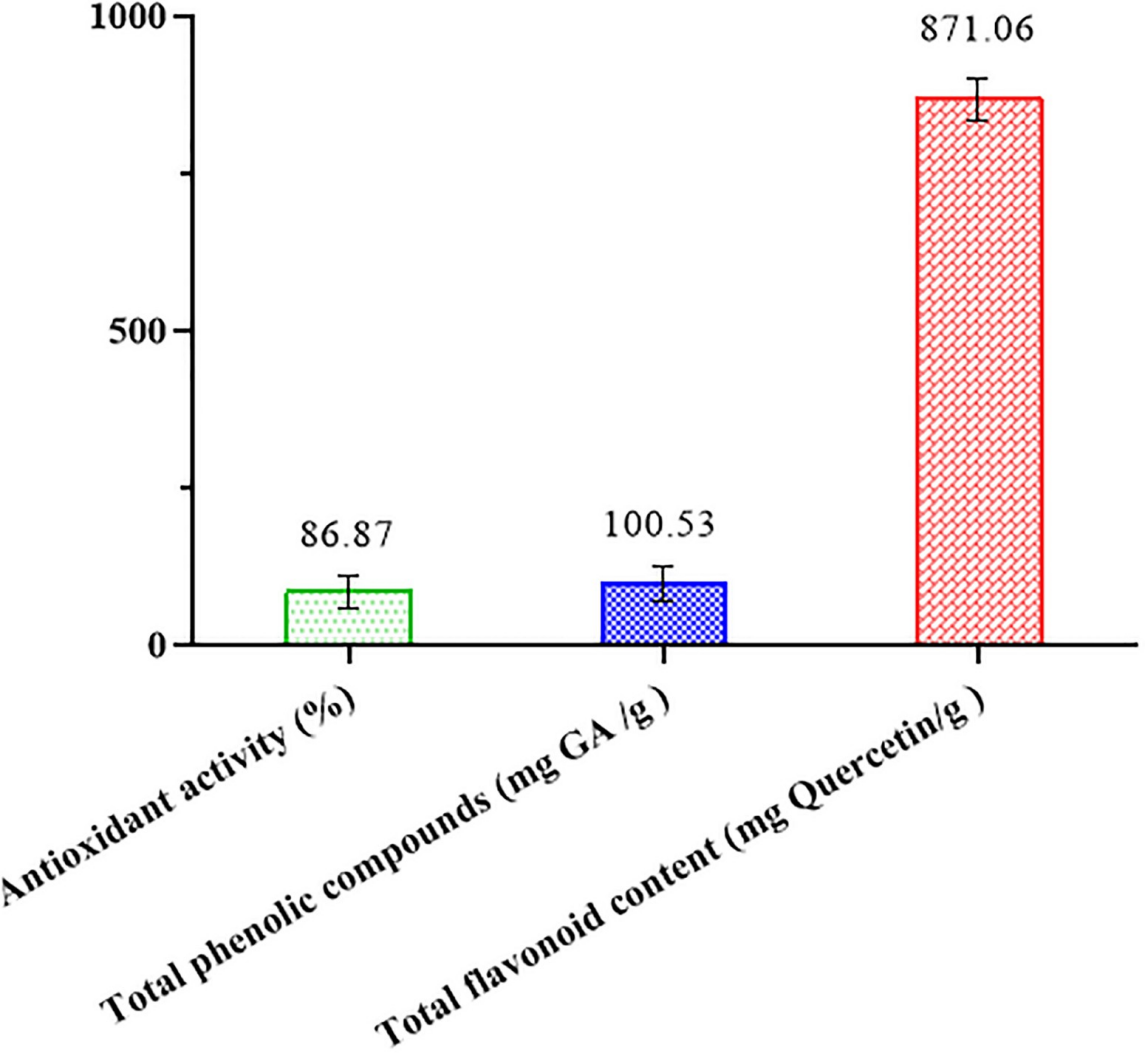
Antioxidant activity, total phenolic compounds and total flavonoid content of black plum peel.

Fresh plums are a rich source of antioxidant activity and phenolic compounds most of them are in the peel of the fruit. It was reported that plum peel contains five times more phenolic compounds per unit of weight than pulp [35]. The results of this study indicated that total phenolic compounds, total flavonoid content and antioxidant activity of black plum peel were 100.53 mg GA /g, 871.062 mg Quercetin/g and 86.87% and respectively (Fig 1). Accordingly, BPP can be effective as an antioxidant in the improvement of oil quality and stability. Mohammadi-Moghaddam et al. (2020) [27] reported antioxidant activity and total phenolic compounds of BPP puree at 105.91 (mg/g GA) and 88.59%, respectively. Stacewicz-Sapuntzakis et al. (2001) [35] reported total phenolic compounds for fresh, dried and juice of plum 111 mg/100g, 184 mg/100g and 44 mg/100g, respectively. According to the results of this research, most of the phenolic compounds in plums are related to neochlorogenic and chlorogenic acids.

#### Peroxide value (PV)

**Fig 2:**
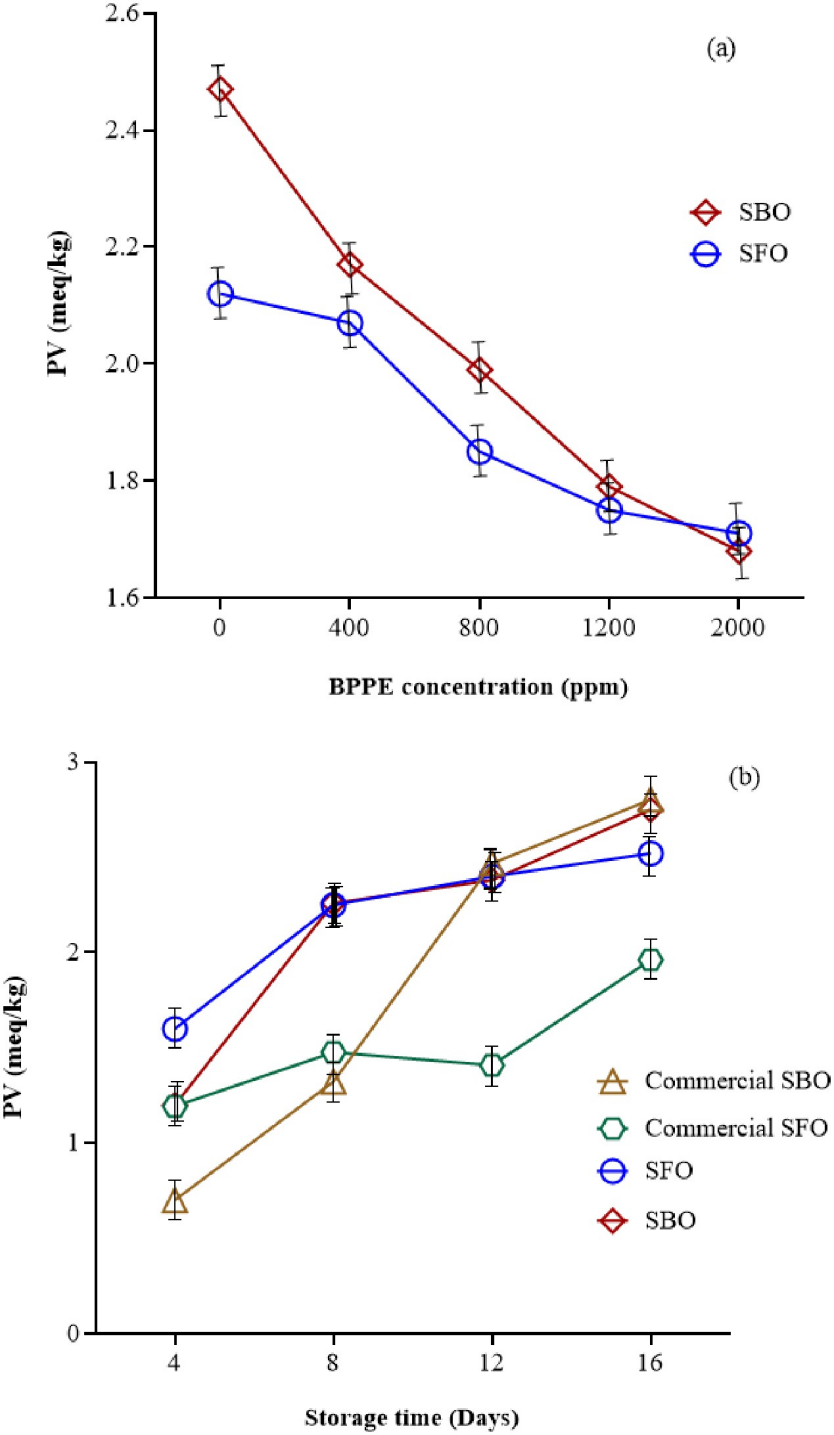
The effect of a) BPPE concentration* and b) storage time* on PV of SBO and SFO. (BPPE: Black plum peel extract, Soy: Soybean oil, Sun: Sunflower oil, CSoy: Commercial soybean oil, CSun: Commercial sunflower oil) *(P≤0.05).

Fig 2a illustrates the peroxide value (PV) of SBO and SFO at different concentrations of BPPE. Increasing the concentration of BPPE decreased the PV (P≤0.05) that show BPPE has been able to show its antioxidant role well. As can be seen in Fig 2b, increasing the storage days in raw and commercial oils caused to increase PV (P≤0.05). The increasing slope of PV for commercial SBO was much more than others. For the samples containing BPPE, the amount of PV was very near from day 12 onwards. Peroxide value for SBO and SFO after storage up to 16 days was in the range of 0.95 to 2.84 meqO2/kg and 1.11 to 2.89 meqO2/kg, respectively. This finding of the current study is consistent with those of other studies [7, 9, 10, 20, 36], Asnaashari et al. (2015) studied the effect of *R*. *fruticosus* leaves extract in stabilizing sunflower oil during accelerated storage. Their results showed the usage of R. fruticosus leaves extract could reduce the peroxide value and increase the oxidative stability of sunflower oil.

#### Free fatty acid content (FFA)

**Fig 3:**
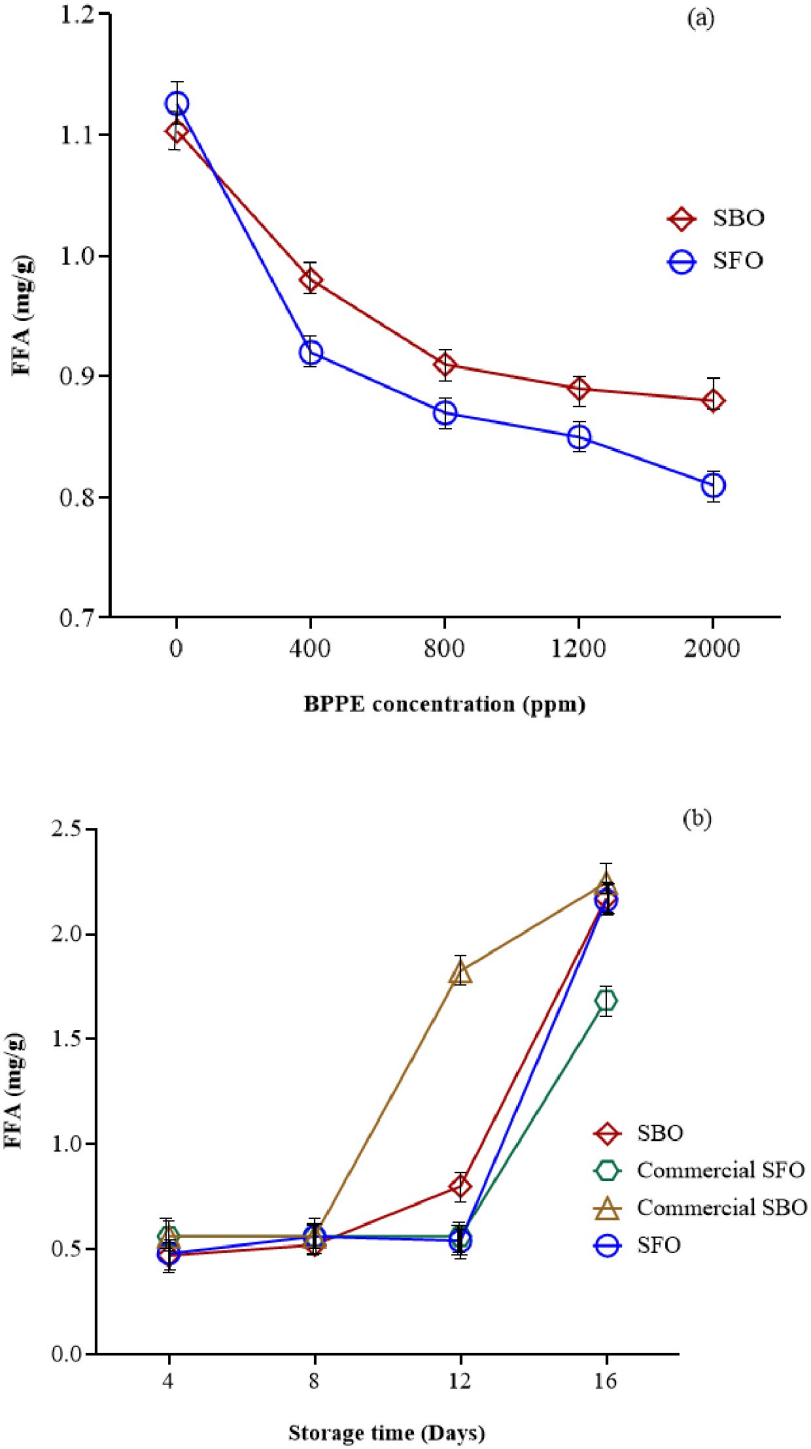
The effect of a) BPPE concentration* and b) storage time* on FFA of SBO and SFO. (BPPE: Black plum peel extract, Soy: Soybean oil, Sun: Sunflower oil, CSoy: Commercial soybean oil, CSun: Commercial sunflower oil) *(P≤0.05).

The formation of free fatty acids could be an important parameter of rancidity in food products. FFAs are formed due to the hydrolysis of triglycerides and can get upgraded by the reaction of oil with moisture [37]. The results obtained from the analysis of FFA are shown in Fig 3a and 3b. There was a non-linear negative correlation between FFA and BPPE concentration (Fig 3a). It is also observed that these changes are similar in soybean and sunflower oils. Further analysis showed that the amount of FFA increased over time (P≤0.05). Although, there was no significant difference until day12. But as shown in Fig 3b, from day 12 to day 16, the amount of FFA increased significantly. Similar to Fig 2b, the FFA in commercial SBO showed a greater slope than others. FFA for SBO and SFO, after storage up to 16 days was in the range of 0.41 to 2.62 mg/g and 0.16 to 2.25 mg/g, respectively. Iqbal and Bhanger (2007) [37] studied the stabilization of SFO by garlic acid extract at 185 °C, for 24 days. FFA content was increased during storage time. Similar results were obtained by adding the ginger extract to SFO. The FAA of the oil showed a significant decrease compared to the control sample at 45°C for 25 days [38]. Drinić, Z., et al. (2020) [9] studied the effect of pomegranate peel extract on the oxidative stability of pomegranate seed oil. There was no significant difference between the samples during the storage time and the acid value in all samples of pomegranate seed oil was low.

#### Tiobarbituric acid (TBA)

**Fig 4:**
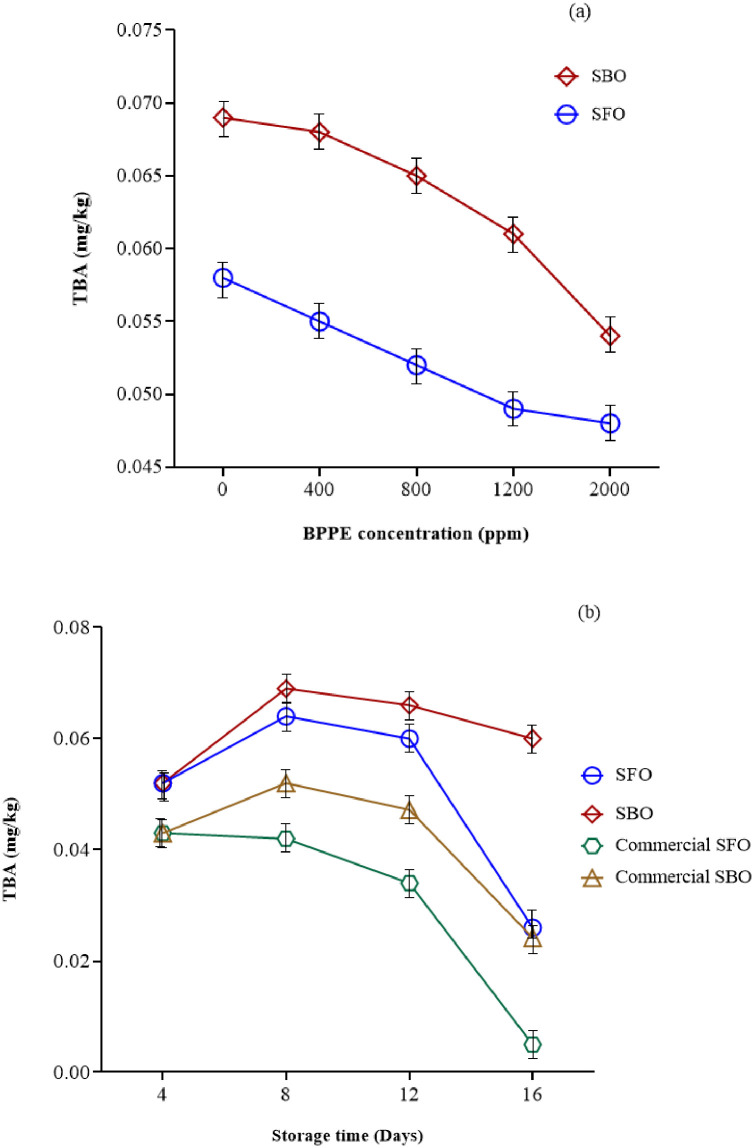
The effect of a) BPPE concentration* and b) storage time* on TBA of SBO and SFO. (BPPE: Black plum peel extract, Soy: Soybean oil, Sun: Sunflower oil, CSoy: Commercial soybean oil, CSun: Commercial sunflower oil) *(P≤0.05).

The PV alone does not indicate the oxidation of the oil because it is an indicator of the presence of primary oxidation products and does not show the production of oxidation by-products. Therefore, the existence of a test that shows the development of oxidation and the production of by-products of this reaction is necessary [10]. The TBA test is used to determine the amount of malondialdehyde as the major secondary by-product of lipid oxidation in food products. Based on results indicated in Fig 4a, TBA values increased during the storage time from 4 days to 8 days (P≤0.05). However, beyond 8 days of storage time reduced the TBA values (P≤0.05). Fig 4b shows that TBA values was reduced with increasing the concentration of BPPE (P≤0.05). These results indicate that BPPE has been able to play a suitable role in preventing the production of oxidation by-products. The presence of phenolic compounds in the BPPE causes to delay the oxidation of the oils. These substances prevent the progression of oxidation reactions by inhibiting free radicals and reducing the oxidation of secondary compounds such as malondialdehyde. TBA value for SBO and SFO, after storage for up to 16 days was in the range of 0.04 to 0.08 and 0.02 to 0.07, respectively. This finding is in agreement with other researches [20, 21, 36, 39, 40]. Baştürk, A., et al. (2018) [21] studied the influence of raspberry leaves extract on the oxidative stability of SFO for up to 12 days under heating conditions at 70°C. Totally, the TBA value increased by increasing the storage time. But no regular pattern of increase could be observed for samples.

#### Conjugated dienes (CD)

**Fig 5:**
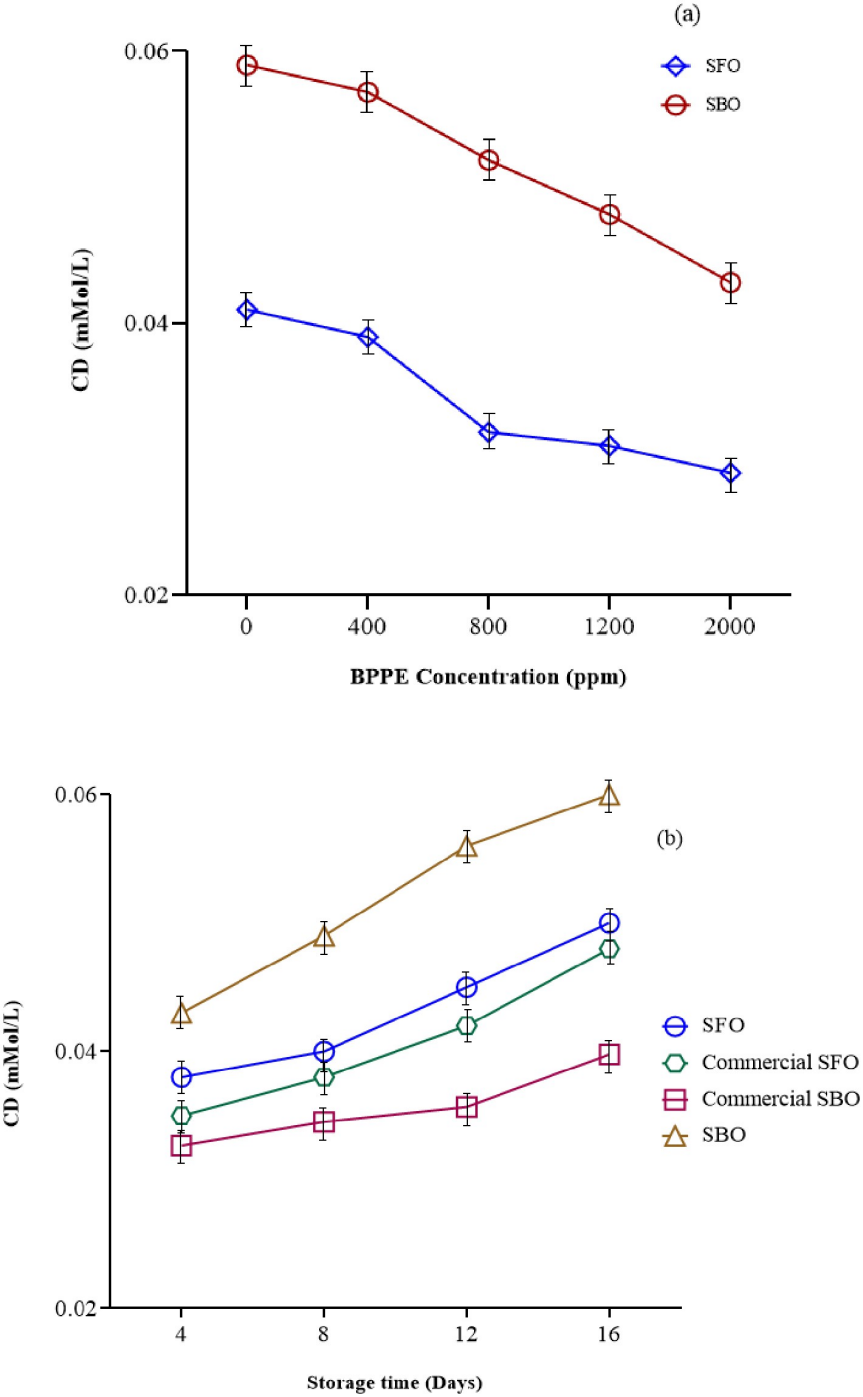
The effect of a) BPPE concentration* and b) storage time* on CD of SBO and SFO. (BPPE: Black plum peel extract, Soy: Soybean oil, Sun: Sunflower oil, CSoy: Commercial soybean oil, CSun: Commercial sunflower oil) *(P≤0.05).

Conjugated dienes are primary products of oxidation that formed almost directly after peroxides. With the development of lipid oxidation, the conjugated dienes are broken down into secondary products. Secondary products can’t absorb UV-visible light forcefully and it causes to decrease in absorbance. As can be seen from Fig 5a, increasing the storage time of oils increased the CD values (P≤0.05), although after day 12 the slope was steeper. Maybe, after this time Diels-Alder mechanism happens and conjugated compounds are polymerized [41]. From Fig 5b, it can see that increasing the concentration of BPPE reduced the CD (P≤0.05) showing BPPE is well able to prevent the formation of conjugated dienes. It seems that the high antioxidant activity of black plum peel extract has been able to reduce the oxidation effect in the oils. CD value for SBO and SFO was in range from 0.01 to 0.08 mMol/L and 0.02 to 0.07 mMol/L, respectively. The present findings seem to be consistent with other researches [21, 36, 37, 39].

### Carbonyl value (CV)

**Fig 6:**
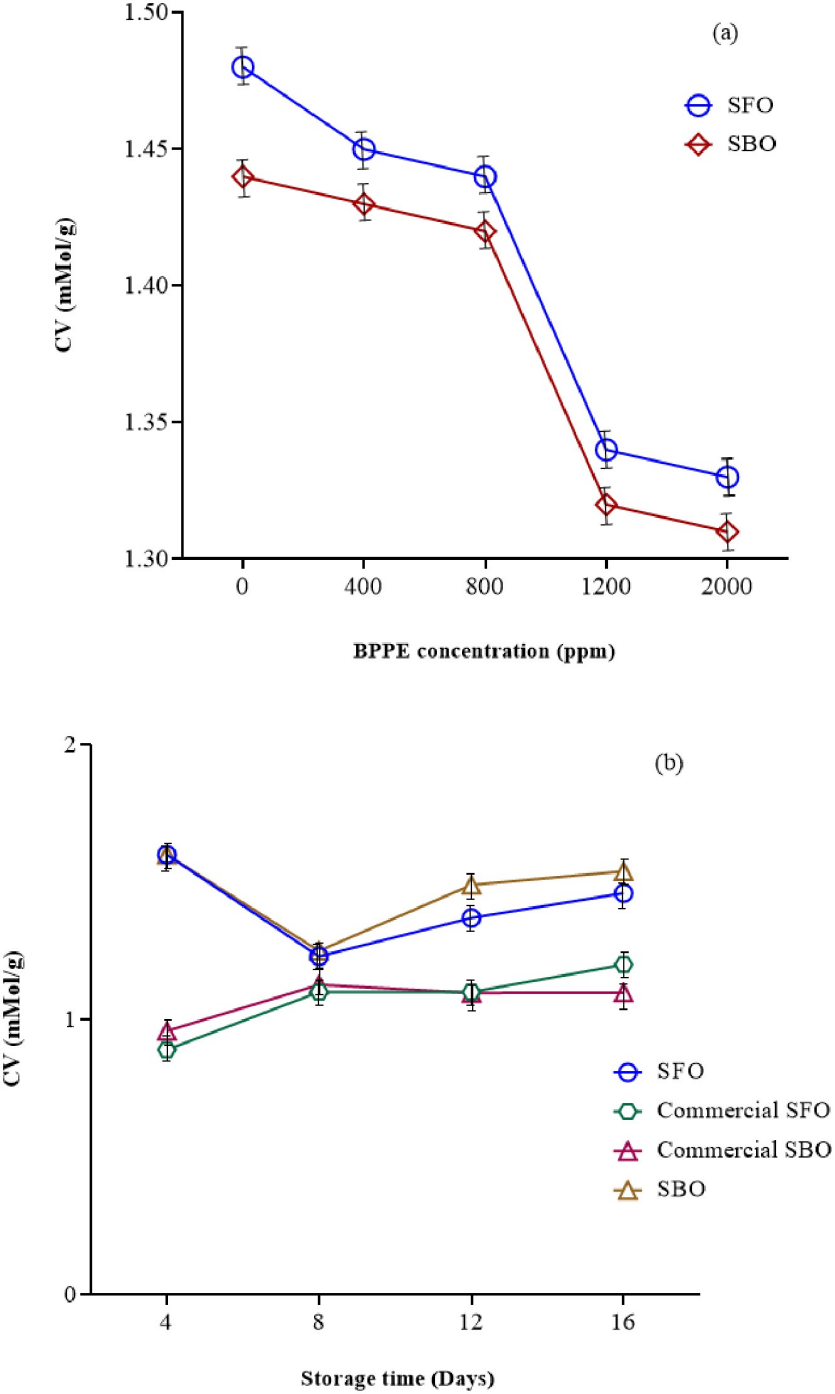
The effect of a) BPPE concentration* and b) storage time** on CV of SBO and SFO. (BPPE: Black plum peel extract, Soy: Soybean oil, Sun: Sunflower oil, CSoy: Commercial soybean oil, CSun: Commercial sunflower oil) *(P≤0.05), ** (P>0.05).

CV is a good indicator of oxidative changes in oils and fats. Measurement of carbonyl compounds is very important in determining the quality of heated and fried oils. Since, these compounds participate in creating unpleasant flavors and reduce the quality of frying oils [42]. The results obtained from the ANOVA showed that increasing the BPPE increased the CV for SBO and SFO (P≤0.05) (Fig 6a). Fig 6b provides the effect of storage time on the CV of SBO and SFO. As can be seen, the CV of oils increased during the storage time, although none of these differences was statistically significant (P>0.05). The CV is reported to be 0.5 to 2 mMol/g for well-refined oils [42]. Therefore, the oils used in this study had good quality and the amount of CV during the thermal process was less than 2. It has also been reported that if the amount of CV is more than 50, that oil is unusable and the time it takes for the oil to reach this stage is called the critical time [42]. In this study, the critical time for SBO and SFO did not occur after 16 days. So, both types of oils used in this study had good quality during the storage time. The CV value for SBO and SFO was in the range of 0.93 to 2.31 mMol/g and 1.17 to 1.83 mMol/g, respectively.

## Conclusion

Because of the negative properties of synthetic antioxidants on health, the possibility of replacing black plum peel extract (BPPE) as an alternative to commercial antioxidants in soybean oil (SBO) and sunflower oil (SFO) was investigated. The high amount of antioxidants and phenolic compounds of BPP could be introduced as a suitable substance to increase the oxidative stability of oils. According to the results, the usage of BPPE in SBO and SFO causes reduced PV, FFA, TBA, CD and CV. The highest tested concentration of BPPE to increase the oxidative stability of SBO and SFO was 2000 ppm. In addition, storage of oils for 16 days at 60 °C, reduced the oxidative stability of oils. The oxidative stability parameters for commercial oils and samples containing BPPE were near each other and in an acceptable range. It means that black plum peel has been able to make antioxidant activity similar to commercial antioxidants in SBO and SFO oils. Black plum peel extract could have a significant positive effect (P<0.05) on improvement of the quality and stability parameters of soybean oil and sunflower oil. The use of BPP, in addition to its health effects, can play an important role in reducing environmental pollution and the use of plum processing by-products.

## Supporting information

S1 Data (XLSX)
